# Coping strategies and mental wellbeing among TVET students: the moderating role of self-efficacy

**DOI:** 10.3389/fpsyg.2026.1951147

**Published:** 2026-10-20

**Authors:** Naomi Odira Owuor, Bettina F. Piko

**Affiliations:** 1 Doctoral School of Education, University of Szeged, Szeged, Hungary; 2 Department of Behavioral Sciences, University of Szeged, Szeged, Hungary

**Keywords:** coping strategies, mental wellbeing, moderation, self-efficacy, TVET

## Abstract

**Introduction:**

Mental wellbeing is central to students' academic development and psychological functioning. However, coping strategies and self-efficacy as psychological resources that promote mental wellbeing has received limited attention, within technical and vocational education and training (TVET). Premised on the transactional theory of stress and coping, this study examined the moderating role of general self-efficacy in the relation between coping strategies and mental wellbeing among TVET trainees.

**Methods:**

A cross-sectional survey of 1,937 trainees from 239 institutions was conducted using the Brief Coping Orientation to Problems Experienced Inventory, the General Self-Efficacy Scale, and the Warwick-Edinburgh Mental Wellbeing Scale, with data analyzed through Pearson correlations and Hayes's PROCESS macro (Model 1). Confirmatory factor analysis was used to establish the structure of the coping measure.

**Results:**

A two-factor structure distinguishing approach from avoidant coping was supported. Approach coping was positively associated with mental wellbeing (*r* = 0.589), as was general self-efficacy, which showed the strongest association (*r* = 0.750); avoidant coping showed a negligible bivariate association but a significant negative association when approach coping was controlled. Self-efficacy significantly moderated the relation between approach coping and mental wellbeing (Δ*R*^2^ = 0.008, *p* < 0.001), with a negative interaction term. The wellbeing benefit of approach coping was strongest among trainees with lower self-efficacy (*B* = 0.296) and weakened as self-efficacy increased (*B* = 0.133), though it remained significant across the full range of self-efficacy scores. The corresponding interaction for avoidant coping was not significant.

**Discussion:**

These findings suggest that self-efficacy attenuates rather than replaces the contribution of coping to wellbeing, with engagement-oriented coping most beneficial for trainees with lower self-efficacy. Although the moderation effect was modest in magnitude, the findings highlight the value of self-efficacy enhancement and targeted coping-skills support within TVET institutions.

## 1 Introduction

Recent years have seen an increased research focus on mental wellbeing within educational settings. From a positive psychology perspective, mental wellbeing reflects an individual's ability to meet basic social needs using available resources and experiences of happiness, flourishing, and self-satisfaction (Kim and Lindeman, 2020; Norwich et al., 2022). Beyond the absence of psychological distress, mental wellbeing reflects positive functioning, emotional stability, and the capacity to effectively manage academic pressures (Gore, 2025; Kim and Lindeman, 2020). Research has consistently demonstrated the close link between students' psychological functioning and their academic experiences, sense of control and purpose, emotional balance, self-efficacy, and life satisfaction (Cherewick et al., 2024; Diaz et al., 2026; Johnson and Lester, 2022; Lazarewicz et al., 2024; Rana et al., 2023; Tarimo et al., 2025). In technical and vocational education and training (TVET), trainees are exposed to the growing pace and demands of 21st-century skills requiring pedagogical approaches and curricula to address the needs of the Fourth Industrial Revolution (Azeem et al., 2021). This highlights the need to foster effective coping capacity and self-efficacy to protect the trainee's mental wellbeing as they acquire adequate skills.

Expansive studies have identified several variables that influence students' psychological wellbeing across education levels, suggesting that students appraise and respond to academic demands differently (Diabat and Bellibaş, 2025; Donohue and Bornman, 2021; Le Vigouroux et al., 2025). According to Lazarus and Folkman's (1984) transactional theory of stress and coping, mental wellbeing is a dynamic outcome of individuals' cognitive appraisal of environmental demands and perceived availability of coping resources to manage such demands. The theory posits that stress does not reside solely in the external event but in individuals' assessment of the demands placed upon them (primary appraisal) and the resources available to respond effectively (secondary appraisal) (Lazarus and Folkman, 1984). In the transactional approach, coping constitutes an individual's cognitive and behavioral effort to respond to environmental and internal demands that are perceived to threaten their overall mental wellbeing (Freire et al., 2020). Simply put, students' coping responses, perceived resources, and personal competencies play critical roles in shaping their psychological outcomes when they confront stressful events.

While coping strategies are widely regarded as a mechanism that promotes psychological adjustment, the inconsistency of findings suggests that their protective effects depend on the availability of personal resources such as self-efficacy (Choi et al., 2026; Freire et al., 2020). Coined by Bandura (1977), self-efficacy refers to an individual's belief in their ability to execute the required behaviors for managing demanding situations that promote persistence and adaptive coping responses. Expectations of self-efficacy demonstrate whether individuals can initiate coping behaviors, and to what extent and for how long, when confronted with challenges and adverse experiences (Bandura, 1977). Considering the demanding TVET curriculum and heightened expectation to acquire industry-ready skills that match labor market demands, alongside project-based training models designed to reduce the skills gap (Akinlade et al., 2025; UNESCO, 2023), trainees often experience academic pressure and may view these demands as pressure-inducing. As highlighted by Solberg et al. (2023) and Achuodho and Piko (2026), TVET trainees experience vocational identity and self-efficacy, among other factors, which may affect their academic achievement, psychological functioning, and overall mental wellbeing. Sustained participation in challenging competency-based training environments may foster mastery experiences that progressively reinforce self-efficacy and attenuate avoidance-oriented coping responses.

Studies have observed that coping strategies may not independently drive mental wellbeing; instead, their effectiveness may depend not only on students' perceived capability to address academic challenges but also on their educational and geographical context and cultural orientation (Dorado Barbé et al., 2025; Waterhouse and Samra, 2025). In vocational systems, coping strategies are shaped by contextual factors that reinforce or undermine self-efficacy among trainees. A study in China revealed a strong association between self-efficacy and the acquisition of practical skills and experiences in vocational-based training (Fu, 2024). Further on these findings, Fu (2024) suggested the central role of supportive systems such as teachers in building learners' self-confidence especially through encouragement and constructive feedback. In Africa, a study demonstrated a link between self-efficacy and coping strategies, indicating that learners who reported greater confidence in addressing academic challenges were more likely to implement adaptive coping strategies through supportive networks, which in turn promote mental wellbeing (Akeredolu et al., 2025).

Understanding this conditional relation is particularly relevant in Kenya's TVET settings, where trainees often encounter competency-based and assessment training demands. Despite extensive global and regional evidence on the roles of coping strategies and self-efficacy on mental wellbeing, empirical research has hardly focused on the TVET context in Kenya. Nevertheless, recent efforts have started addressing this gap by demonstrating how perceived academic support mediates academic stress and mental wellbeing (Owuor et al., 2026) and how academic resilience mediates perceived support and mental wellbeing (Owuor and Pikó, 2026) for Kenyan TVET trainees. However, these studies have focused on supportive and resilience-based pathways rather than the interplay between coping strategies and self-efficacy. This gap is noteworthy given evidence of self-efficacy as a key resource that influences students' navigation of the practice-oriented and labor-driven training environment, potentially modifying impact of coping strategies on mental wellbeing. Therefore, this study aims to investigate how self-efficacy moderates the relation between coping strategies and mental wellbeing among TVET trainees in Kenya.

According to the transactional theory of stress and coping, interactions between individuals' behavioral responses and personal beliefs influence psychological adaptation within their environment (Lazarus and Folkman, 1984). It posits that individuals exhibit coping behaviors to actively regulate internal states when encountering demands and that the effectiveness of such behaviors determines subsequent psychological adjustment (Nathan and Folkman, 2011). Within a learning environment, learners' wellbeing is defined not only by the presence of coping behaviors but also by the functioning of these behaviors under different levels of perceived competence.

At the core of the transactional theory of stress and coping lies the idea of cognitive appraisal, which refers to individuals' assessment of the importance of the demand and of the sufficiency of their personal resources to cope with perceived stressors. Lazarus and Folkman (1984) identified two different sequential phases: primary appraisal, which evaluates the stressor as threatening or challenging, and secondary appraisal, which gauges the sufficiency of available coping resources for managing the demands. It is within this second phase that self-efficacy beliefs become directly significant. Students who perceive themselves as competent and capable are more likely to view academic demands as manageable, mobilizing active coping efforts that sustain psychological functioning, whereas those who doubt their capabilities are more likely to consider the same demands as threatening, exceeding available resources and leading to psychological strain (Chongjin et al., 2025).

In terms of conceptualizing coping, this study adopted the Brief Coping Orientation to Problems Experienced (Brief-COPE) framework (Carver, 1997). Carver's original organization distinguishes problem-focused, emotion-focused, and avoidant coping, although the higher-order organization of these aspects has varied considerably in different populations and cultural settings. A broader and more consistently replicated distinction was proposed by Roth and Cohen (1986), who differentiated approach and avoidant coping. Approach coping entails situations in which the individual engages with the stressor or with the emotions it generates, while avoidant coping involves situations whereby the individual disengages from it. Approach-oriented strategies are associated with secondary appraisal processes in which individuals actively evaluate stressors as manageable and solvable using available resources, and they include both instrumental responses such as planning and active coping and emotion-regulatory responses such as seeking support, positive reframing, and acceptance (Roth and Cohen, 1986). Avoidant coping reflects disengagement from the stressor through denial, substance use, and behavioral disengagement and is consistently associated with poor psychological outcomes (Le Vigouroux et al., 2025; MacIntyre et al., 2020; Ruiz-Camacho et al., 2025). In the TVET context, where trainees face the combined pressures of competency-based training and assessment, practical skill acquisition, and intense training to fit labor market demands, approach-oriented coping strategies are expected to promote better mental wellbeing, whereas disengagement and denial tend to weaken mental wellbeing and hinder positive academic adjustment.

Studies suggest that coping styles are often stronger among students who perceive themselves as capable of managing academic demands and navigating related challenges, effectively leading to a relaxed psychological adjustment (Addy et al., 2021; Kwon, 2025; Pan et al., 2024; Waterhouse and Samra, 2025). Trainees' response to stress is shaped not only by the presence of stressors but also by their appraisal of available personal and social resources for managing such demands (Lazarus and Folkman, 1984), which strongly correlates with mental wellbeing outcomes such as anxiety, depression, and harmful behaviors (Freire et al., 2020). Consequently, coping research has increasingly examined the suitability of specific coping strategies by exploring their positive and negative effects on trainees. For example, Moreno-Montero et al. (2024) and Chong (2026) suggested that adaptive coping strategies such as problem-solving predicted psychological wellbeing; hence, trainees who implement such strategies reported lower levels of depression, anxiety, and burnout. Similarly, Theodoratou et al. (2023) demonstrated that trainees who employed emotion-focused coping understood and accepted stressful situations by adapting to emotional expressions to deal with academic demands. Conversely, maladaptive coping such as denial, procrastination, and disengagement predicted psychological distress (Rodríguez et al., 2026).

TVET trainees undergo rigorous competency-based training and formative assessments, which significantly elevate academic pressure. Unlike their higher-education counterparts, TVET trainees must both acquire technical expertise and meet workplace-readiness requirements throughout their training period, placing a dual burden and compounding the stress appraisal and pressure on individual coping resources. Hence, coping strategies are likely to play a significant role in shaping trainees' mental wellbeing. This study investigated the relation between coping strategies and general self-efficacy and mental wellbeing in TVET trainees, a population that is particularly vulnerable to stress yet under-researched.

Based on the reviewed literature, we have formulated the following hypotheses:

**H1:** We hypothesize a significant positive association exists between coping strategies and mental wellbeing among TVET trainees. The literature has empirically linked self-efficacy to coping strategies such as problem-solving abilities, emotional expression, and cognitive skills (Freire et al., 2020; Morales and Pérez-Mármol, 2019). The extent of trainees' belief in their ability to cope, manage their abilities to successfully accomplish goals, and view challenges as tasks to accomplish rather than avoid significantly affects their academic performance and mental wellbeing outcomes (Cherewick et al., 2024).
**H2:** We hypothesize a significant positive association between coping strategies and general self-efficacy. Efficacy beliefs are directly linked to psychological adjustment. Individuals who believe they can address challenges experience greater emotional adjustment and life satisfaction as well as lower psychological distress. Moreover, trainees with higher self-efficacy tend to seek social and instrumental support, thus improving their psychological adjustments and academic outcomes and creating a flexible coping mechanism, consequently reducing mental distress (Sun et al., 2025). Conversely, trainees with lower self-efficacy, struggle to manage increased academic demands, often employing maladaptive coping mechanisms such as procrastination, denial, and disengagement (Choi et al., 2026). This suggests the pivotal role of self-efficacy in contributing to trainees' mental wellbeing.
**H3** : We hypothesize a significant positive association exists between general self-efficacy and mental wellbeing. Extensive literature has observed the association between efficacy beliefs and psychological adjustment (Freire et al., 2019; Morales and Pérez-Mármol, 2019). Trainees who believe in their capacity to manage academic stressors tend to report greater emotional adjustment, persistence in the face of difficulty, and lower psychological distress (Grotan et al., 2019; Akeredolu et al., 2025).

The transactional theory of stress and coping posits that the cognitive appraisal framework involves behavioral responses, implying that coping behaviors do not necessarily have similar effects; instead, their contribution depends on the appraisal resources an individual already has. Where efficacy beliefs are strong, they provide much of the appraisal and persistence capacity that coping would otherwise provide, so the additional contribution of coping-engagement is expected to be weaker.

**H4:** Finally, we hypothesize that general self-efficacy significantly moderates the relation between coping strategies and mental wellbeing, such that as self-efficacy increases, the positive association between coping and mental wellbeing decreases (Choi et al., 2026; Freire et al., 2020). For trainees with limited self-efficacy beliefs, coping engagement is expected to have a stronger weight.

Together, these hypotheses propose that general self-efficacy moderates the relation between coping strategies and mental wellbeing such that the positive association between coping and wellbeing is weaker among trainees with stronger efficacy beliefs. Figure 1 illustrates the hypothesized model.

**Figure 1:**
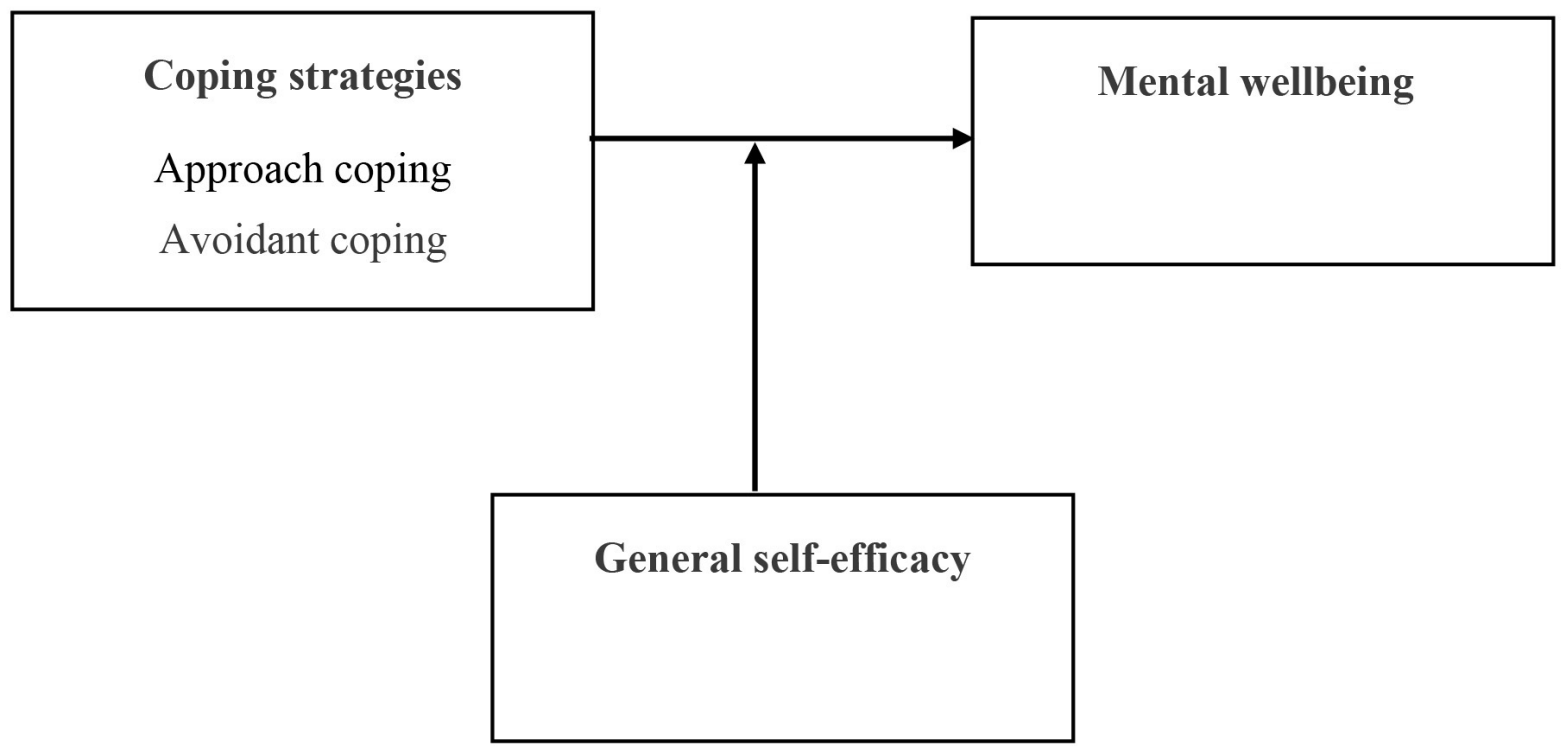
Hypothesized model of general self-efficacy as a moderator of the relation between coping strategies and mental wellbeing.

## 2 Materials and methods

### 2.1 Study design and participants

This cross-sectional study conducted a survey that investigates the moderating role of general self-efficacy in the relation between coping strategies and mental wellbeing. It involved 239 TVET institutions with a population of 400,000 trainees across all regions in Kenya (Republic of Kenya, 2024). Multistage sampling was performed in which institutions were first stratified into five geographical regions: Nairobi, Mount Kenya, Coast, Western, and Rift Valley. These institutions were then cluster-sampled in each region, and the trainees were simple-random-sampled in the selected institutions. These institutions were then cluster sampled in each region, while the trainees were simple random sampled within these institutions. The target sample size was determined on two criteria. First, for the population of trainees estimated at 400,000 (Republic of Kenya, 2024), the use of Cochran's (1977) formula for large populations suggests a minimum of 384 respondents at the 95% confidence level and ±5% margin of error. Second, the study's main analyses involved moderation and interaction effects which tend to be small in non-experimental field research settings and need larger samples to be detected, an a priori power analysis was conducted using G^*^Power 3.1 (Faul et al., 2009; Shieh, 2009). An increase in *R*^2^ caused by one interaction term in multiple regression could be detected as small (*f*^2^ = 0.01) at α = 0.05 and power of 0.95 for 1,302 participants. The target participants were therefore set above this threshold yielding a final analytical sample of 1,937 TVET trainees (57.7% males, 42.3% females; age range 18–37 years; *M* = 22.83, *SD* = 3.15). These ages reflected the broad developmental range among trainees in technical and vocational education environments, who are attracted to the field's flexible entry criteria and diverse educational pathways. The highest percentage of respondents were in year 2 of their enrollment (40.3%), followed by those in years 1 (36.1%) and 3 (23.6%).

In terms of educational level, the responses were spread across level 3 (18.3%), level 4 (25.9%), level 5 (21.8%), and level 6 (33.9%), while for institution type, the majority of trainees were enrolled in national polytechnic (54%), followed by technical training institutes (25.0%) and technical and vocational colleges (21.0%). The majority of the courses were technical (73.9%), while the rest were business programs (26.1%). An overwhelming majority of participants were from low-income households (79.1%), while a small proportion reported medium-income backgrounds (17.5%), and a smaller minority came from high-income families (3.4%). These sociodemographic characteristics were reported to describe the sample. To assess the robustness of psychological associations to demographic variation, they were additionally entered as covariates in supplementary moderation models as reported in Section 3.4. Table 1 summarizes these demographics.

**Table 1:** The participants' sociodemographic characteristics.

Variable	Category	*N*	%
Age (years)	M = 22.83, SD = 3.15	_	_
Gender	Male	1,118	57.7
	Female	819	42.3
Year of study	First year	699	36.1
	Second year	781	40.3
	Thirdyear	457	23.6
Level of study	Level 3	354	18.3
	Level 4	502	25.9
	Level 5	422	21.8
	Level 6	659	33.9
Course classification	Technical	1,431	73.9
	Business	506	26.1
Institution type	National polytechnic	1,046	54.0
	Technical training institute	484	25.0
	Technical and vocational college	407	21.0
Family Income (Monthly)	Low ( ≤ KSh 14,000)	1,534	79.1
	Medium (KSh 14,001–56,000)	339	17.5
	High (≥ KSh 56,000)	66	3.4

These variables were entered as covariates in supplementary moderation model.

### 2.2 Instruments

Coping strategies were measured using the Brief-COPE Inventory (Carver, 1997), a widely used 28-item self-report instrument for assessing adaptive and maladaptive coping mechanisms across diverse populations. This tool consists of problem-focused coping, emotion-focused coping, and avoidance subscales (Carver, 1997), although its higher-order structure was tested empirically in the present sample. Participants indicated how often they engaged in each coping behavior on a Likert scale from 1 (“I haven't been doing this at all”) to 4 (“I've been doing this a lot”). The Brief-COPE Inventory was selected as it captures the multidimensional nature of coping, which is particularly salient within the TVET context, where trainees navigate competency-based training and instructional load stressors with limited training resources as well as the heightened academic demands of industry readiness. Two items were excluded prior to analysis; one duplicated the wording of another item, and one did not correspond to any Brief-COPE facet, leaving 26 items.

Since the higher order structure of the Brief-COPE appears to differ across cultural and educational environments, we tested the structure of the Brief-COPE in the present sample using confirmatory factor analysis (CFA) with robust maximum likelihood estimation (MLR) in Mplus 8.3. Four models were compared. The three-factor structure separating problem-focused, emotion-focused and avoidant coping fitted poorly (CFI = 0.807, TLI = 0.788, RMSEA = 0.075, SRMR = 0.123) and resulted in a non-positive-definite latent covariance matrix, with the problem-focused and emotion-focused subscales correlating at 0.982. The two-factor approach-avoidance model retaining the original item assignments also fitted inadequately (CFI = 0.842, TLI = 0.827), and the three-factor with the self-distraction items reassigned to the problem-focused factor improved fit (CFI = 0.907, TLI = 0.897), but produced inadmissible results with problem-focused and emotion-focused coping correlating at 0.985. Only the two-factor model distinguishing approach from avoidant coping, with the self-distraction items loading on the approach factor, produced an admissible solution with acceptable fit (CFI = 0.897, TLI = 0.888, RMSEA = 0.055, SRMR = 0.083) and clearly differentiated factors (*r* = 0.410), which was the retained structure. However, we should note here that these values are suboptimal in terms of the first two indicators, compared to the conventional thresholds for good fit (≥0.95) and for marginal fit (≥0.90). It was retained because it was the only admissible solution among the four models compared, the remaining specifications producing non-positive-definite latent covariance matrices.

The inter-factor correlation between the two-factor model dropped sharply from 0.924 (Model 2) to 0.410 (Model 4). This is attributed to the disproportionate influence of the two self-distraction items on the avoidance factor. In the former model, the two self-distraction items loaded most strongly on that factor (0.694 and 0.705), while the two substance-use items loaded weakly (0.165 and 0.187) accounting for less than 4% of their variance. The avoidance factor was therefore defined largely by self-distraction, which in this sample functions as an engagement-oriented strategy, producing the high correlation with approach coping. After the self-distraction items were reassigned, the factor was defined by denial, substance use and behavioral disengagement with loadings distributed more evenly (0.554–0.674) and the substance-use items explaining far more variance (R^2^ rising from 0.027 and 0.035 to 0.341 and 0.363). The reduced correlation therefore indicates a more clearly defined avoidance construct rather than an arbitrary consequence of relocating two items.

Approach coping (20 items) comprises active coping, planning, instrumental and emotional support, positive reframing, acceptance, religion, humor, venting, self-blame, and self-distraction (α = 0.934). Avoidant coping (6 items) comprises denial, substance use and behavioral disengagement (α = 0.793). Following the exclusion of the two items noted above, planning and self-blame facets are each represented by a single item. The results of the confirmatory factor analysis are presented in full in Table 2, and mapping of all administered items, their Brief-COPE facets and retained dimensions are provided in Supplementary Table S1.

**Table 2:** Confirmatory factor analysis of the Brief-COPE: comparison of competing models.

Model	χ^2^	df	CFI	TLI	RMSEA [90% CI]	SRMR	AIC	BIC	Factor correlation
1. Three-factor (original)^a^	3534.25	296	0.807	0.788	0.075 [0.073, 0.077]	0.123	134520.54	134971.62	0.982 (PFC–EFC)^b^
2. Two-factor (original items)^c^	2948.98	297	0.842	0.827	0.068 [0.066, 0.070]	0.084	133860.62	134306.13	0.924
3. Three-factor (self-distraction reassigned)^d^	1857.49	295	0.907	0.897	0.052 [0.050, 0.055]	0.079	132414.18	132870.83	0.985 (PFC–EFC)^b^
4. Two-factor (self-distraction reassigned)^e^	2015.05	297	0.897	0.888	0.055 [0.052, 0.057]	0.083	132613.67	133059.18	0.410

*N* = 1,937, Estimator = robust maximum likelihood (MLR), Mplus 8.3. All χ^2^ values *p* < 0.001. Model 4 was retained. ^a^Problem-focused, emotion-focused, and avoidant coping as specified by Carver (1997). ^b^Solution inadmissible: latent covariance matrix not positive definite. ^c^Approach coping (problem- and emotion-focused combined) vs. avoidant coping, retaining original item assignments. ^d^Self-distraction items assigned to problem-focused coping. ^e^Self-distraction items assigned to approach coping; residual correlation freed between the two substance-use items.

General self-efficacy was measured by the General Self-Efficacy Scale (GSE) (Schwarzer and Jerusalem, 1995). This is a well-established and widely used instrument that measures self-efficacy. Self-efficacy refers to the certainty that one can achieve their desired goals and objectives, a crucial aspect for nurturing resilience and ongoing motivation in educational environments (Lopez-Garrido, 2025), including TVET trainees. The GSE contains 10 items rated on a Likert scale from 1 (not at all true) to 4 (exactly true), with higher composite scores suggesting more positive self-efficacy beliefs. In this study, the GSE allowed for a finer-grained interpretation of trainees' perceptions of their ability to address academic and occupational demands. The GSE had a Cronbach's alpha of 0.880, well above the 0.70 threshold for psychological research and in good agreement with Schwarzer and Jerusalem's (1995) reliability estimates from various international samples.

For assessing mental wellbeing, this study applied the Warwick-Edinburgh Mental Wellbeing Scale (WEMWBS), originally developed by Tennant et al. (2007) and subsequently validated by Smith et al. (2017). It contains 14 items rated on a Likert scale from 1 (none of the time) to 5 (all of the time), reflecting two key components of mental wellbeing: positive affect and positive psychological functioning. The scale's relevance to the Kenyan TVET context lies in its ability to encapsulate the “real” psychological functioning and self-efficacy of trainees as they experience academic stressors and of teachers/trainers as they manage teaching demands, a central concern of this study regarding the contributory role of academic factors in mental wellbeing. Statements on this scale include “I've been feeling relaxed,” “I've been dealing with problems well,” “I've been feeling close to other people,” and “I've been feeling loved.” Moreover, the brevity and conceptual simplicity of the WEMWBS make it suitable for all levels of study, especially in TVET institutions, which often enroll a high number of students with different learning abilities. The WEMWBS had an internal consistency of α = 0.91 for the present sample.

Since general self-efficacy and mental wellbeing were substantially correlated in this sample, confirmatory factor analysis was performed to examine discriminant validity. Two-factor model specifying self-efficacy and mental wellbeing as distinct constructs fitted the data well (CFI = 0.960, TLI = 0.956, RMSEA = 0.035, SRMR = 0.029) and considerable better than a single-factor alternative (CFI = 0.913, TLI = 0.905, RMSEA = 0.052, SRMR = 0.043; ΔCFI = 0.047, ΔAIC = 976). The latent correlation between the two constructs was 0.843, indicating that they are related but distinct.

### 2.3 Procedure

The survey was conducted and data collected through an online platform (Google Forms) and was accessed through a QR code and link. Before participating, the trainees provided written (electronic) informed consent via a form that explained the purpose of the study, the voluntary nature of participation, confidentiality, and the freedom to withdraw with no consequences. This study followed the principles of the Declaration of Helsinki and received ethical clearance from the Doctoral School of Education, University of Szeged, Hungary (ref. no. 22/2025) and the National Commission for Science, Technology and Innovation, Kenya (ref. no. 697234). Permission was also obtained from the principals of the TVET institutions involved.

### 2.4 Statistical analysis

Data analysis followed several methodical steps to reveal the relations between the variables and test the hypotheses. For this purpose, this study used an extensive analytical structure. First, descriptive and correlation analyses were performed using SPSS version 26.0 to clarify the characteristics of the variables and explain their associations, respectively. To test the moderating role of GSE, Hayes's PROCESS macro (Model 1) was employed (Hayes, 2022), with a separate moderation model estimated for each coping dimension, while the other dimension entered as a covariate so that each reported effect represents a unique association. Each model was estimated using ordinary least squares regression with heteroscedasticity-consistent standard errors (HC3), and the predictor and moderator were mean-centered before the interaction term was computed to reduce non-essential multicollinearity and facilitate interpretation. Moderation was indicated by a statistically significant interaction (product) term between the coping dimension and GSE. Each coping dimension's conditional effect on mental wellbeing was probed at the 16th, 50th, and 84th percentiles of the moderator, and the Johnson–Neyman technique was applied to identify regions of self-efficacy across which the conditional effect was statistically significant. Model fit was assessed using the coefficient of determination (*R*^2^), the change in *R*^2^ attributable to the interaction (Δ*R*^2^), and F-statistics. An effect was considered statistically significant when its 95% confidence interval did not include zero, with significance evaluated at *p* < 0.05. Models were then re-estimated with age, gender, year of study, level of study, institution type, course classification and family income entered as covariates to assess the robustness of the interaction effects to sociodemographic differences.

## 3 Results

### 3.1 Assumption testing

Before inferential analysis, the statistical assumptions underlying the parametric procedures were examined. Given the large sample (*N* = 1,937), normality was assessed through skewness and kurtosis, which is sensitive to trivial deviations in large sample sizes (Kim, 2013). All values were within acceptable ranges (skewness: −0.618 to 0.189; kurtosis: −0.801 to −0.129; thresholds of ±2 and ±7), and good agreement was achieved between the means and 5% trimmed means, suggesting the negligible influence of outliers. A perusal of the normal P–P plot indicated that the standardized residuals were close to the diagonal, and the residual scatterplot showed that the residuals were centered around zero (*M* = 0.000) with only mild heteroscedasticity. This was done by using HC3 in all moderation models (Hayes, 2022). Multicollinearity diagnostics were all within acceptable limits (Tolerance: 0.492–0.779; VIF: 1.283–2.031), and standardized residuals ranged between −5.969 and 4.837 with extreme values representing less than 1% of the cases. Finally, common method bias was tested using Harman's single-factor test, revealing five components with an eigenvalue >1, which accounted for 51.61% of the variance, with the first unrotated factor explaining only 32.86% of the variance, which is below 50%, showing that common method variance was not a significant threat.

### 3.2 Descriptive statistics and bivariate associations

Table 3 presents the means, standard deviations, and zero-order Pearson correlations for study variables. Approach coping (*M* = 52.50, *SD* = 14.63) scored above its midpoints of 50, while avoidant coping (*M* = 13.00, *SD* = 4.60) fell slightly below its midpoint of 15, indicating comparatively lower engagement in disengagement-oriented strategies. Similarly, GSE scores (*M* = 28.67, *SD* = 7.13) exceeded the scale midpoint of 25, reflecting moderately high self-efficacy among TVET trainees. Mental wellbeing scores (*M* = 46.90, *SD* = 12.55) likewise were above the scale midpoint of 42, suggesting that the sample had moderate to good psychological wellbeing overall.

**Table 3:** Descriptive statistics and correlations for study variables.

Variable	M	SD	1	2	3	4
1. Approach coping	52.50	14.63	—			
2. Avoidant coping	13.00	4.60	0.397^**^	—		
3. Mental wellbeing	46.90	12.55	0.589^**^	0.073^**^	—	
4. General self-efficacy	28.67	7.13	0.608^**^	0.041	0.750^**^	—

*N* = 1,937. M, mean; SD, standard deviation. ^**^p < 0.001. Other correlation *p* < 0.05.

H1 suggests significant positive associations between coping strategies and mental wellbeing among TVET trainees. Pearson product-moment correlations revealed divergent patterns across the two coping dimensions (Table 3). Approach coping demonstrated a strong positive association with mental wellbeing (*r* = 0.589, *p* < 0.001), representing a large effect size according to conventional benchmarks (Cohen, 1988). Avoidant coping, on the other hand, showed a negligible association (*r* = 0.073, *p* = 0.001) with mental wellbeing. Although statistically significant given the large sample, it accounted for less than 1% of the variance in mental wellbeing and is not interpreted as a meaningful positive relation. These findings suggest that approach coping strategy was substantially associated with greater mental wellbeing, whereas avoidant coping showed no meaningful bivariate association among TVET trainees. H1 was therefore partially supported.

According to H2, significant positive associations can be justified between coping strategies and GSE. From the findings, approach coping showed a strong positive association with GSE (*r* = 0.608, *p* < 0.001), representing a large effect size (Cohen, 1988), while avoidant coping, by contrast, showed no significant association with GSE (*r* = 0.041, *p* = 0.073). These results indicate that more active engagement with approach-oriented coping is associated with higher levels of efficacy beliefs, whereas reliance on avoidant strategies was unrelated to efficacy beliefs among TVET trainees, partially supporting H2.

Finally, H3 assumes a significant positive association between GSE and mental wellbeing. Results demonstrate that within the correlation matrix, this correlation was the strongest (*r* = 0.750, *p* < 0.001), highlighting the importance of self-efficacy beliefs as a correlate of psychological wellbeing among TVET trainees. Bandura (1977) theorized that efficacy beliefs serve as a psychological resource for individuals to control their emotional and behavioral reactions to the demands of their environment. Thus, H3 is fully confirmed (Table 3).

### 3.3 Moderation analysis

Moderation analyses were performed using PROCESS Model 1 (Hayes, 2022) for each coping dimension, with other dimension entered as covariant, using HC3 and mean-centered variables. Conditional effects were examined at the 16th, 50th, and 84th percentile values of the moderator, and the Johnson–Neyman technique was applied to establish regions of significance.

For approach coping, the overall model was significant (*R*^2^ = 0.600, *F* = 677.18, *p* < 0.001), explaining 60.0% of the variance in mental wellbeing. Beyond the significant main effects of approach coping (*B* = 0.218, *SE* = 0.026, *t* = 8.347, *p* < 0.001, 95% CI [0.167, 0.269]) and GSE (*B* = 1.029, *SE* = 0.050, *t* = 20.736, *p* < 0.001, 95% CI [0.932, 1.127]), the interaction term was statistically significant and negative (*B* = −0.010, *SE* = 0.002, *t* = −4.711, *p* < 0.001, 95% CI [−0.014, −0.006], Δ*R*^2^ = 0.008, *F* = 22.189, *p* < 0.001). Avoidant coping, entered as a covariate, was significantly and negatively associated with mental wellbeing (*B* = −0.183, *SE* = 0.045, *t* = −4.086, *p* < 0.001). Examination of conditional effects revealed an attenuating moderation pattern; the effect of approach coping on mental wellbeing was strongest at low (16th percentile) levels of self-efficacy (*B* = 0.296, *SE* = 0.031, *p* < 0.001, 95% CI [0.235, 0.357]), moderate at mean (50th percentile) levels (*B* = 0.225, *SE* = 0.026, *p* < 0.001, 95% CI [0.173, 0.276]), and weakest at high (84th percentile) levels (*B* = 0.133, *SE* = 0.031, *p* < 0.001, 95% CI [0.072, 0.195]). Johnson–Neyman analysis showed that the effect of approach coping on mental wellbeing remained statistically significant across the entire observed range of GSE scores, suggesting that active, goal-directed coping constitutes a universally meaningful psychological resource for wellbeing among TVET trainees.

For avoidant coping, the overall model was significant (*R*^2^ = 0.594, *F* = 634.69, *p* < 0.001). Avoidant coping was negatively associated with mental wellbeing (*B* = −0.102, *SE* = 0.045, *t* = −2.256, *p* = 0.024, 95% CI [−0.191, −0.013]), while approach coping, entered as a covariate, remained positively associated with mental wellbeing (*B* = 0.198, *SE* = 0.027, *t* = 7.315, *p* < 0.001). However, the interaction between avoidant coping and GSE did not reach statistical significance (*B* = −0.016, *SE* = 0.009, *t* = −1.888, *p* = 0.059, 95% CI [−0.033, 0.001], Δ*R*^2^ = 0.002, *F* = 3.566*, p* = 0.059). These results indicated that avoidant coping was associated with lower mental wellbeing. Conditional effects were therefore not reported since the interaction was not statistically significant (*p* = 0.059).

To assess whether these findings were attributable to sociodemographic differences, both models were re-estimated with age, gender, year of study, level of study, type of institution, course classification and family income entered as covariates. The approach coping-GSE interaction was essentially unchanged (*B* = −0.010, *SE* = 0.002*, t* = −4.554, *p* < 0.001, Δ*R*^2^ = 0.007), as was the pattern of conditional effects (*B* = 0.321, 0.249, and 0.156 at the 16th, 50th, and 84th percentiles respectively, all *p* < 0.001). Gender (*B* = −2.002, *p* < 0.001), course classification (*B* = −1.266, *p* = 0.001) and age (*B* = −0.148, *p* = 0.005) were among the covariates that significantly associated with mental wellbeing, whereas year of study, level, institution type, and family income were not. The stability of the interaction across specifications indicated that the moderating role of self-efficacy is not accounted for by observed sociodemographic differences.

Collectively, the moderation analyses partially supported H4 (Table 4). A significant negative interaction emerged for approach coping but not for avoidant coping, indicating that GSE operates as a psychological buffer specifically in relation to engagement-oriented strategies, gradually reducing the degree to which mental wellbeing is associated with coping. Trainees with lower self-efficacy beliefs obtained proportionally greater wellbeing benefits from approach coping, whereas those with higher self-efficacy beliefs relied less on coping as a psychological resource (Bandura, 1977; Lazarus and Folkman, 1984).

**Table 4:** General self-efficacy as a moderator of the relation between coping strategies and mental wellbeing.

	Approach coping			Avoidant coping		
Predictor	B	SE	95% CI	B	SE	95% CI
Step 1 – Main effects						
Constant	49.913	0.674	[48.592, 51.234]	36.545	1.476	[33.650, 39.441]
Coping (X)	0.218	0.026	[0.167, 0.269]	−0.102	0.045	[−0.191, −0.013]
GSE (W)	1.029	0.050	[0.932, 1.127]	1.061	0.051	[0.962, 1.161]
Covariate	−0.183	0.045	[−0.270, −0.095]	0.198	0.027	[0.145, 0.251]
Step 2 – Interaction						
Coping × GSE	−0.010	0.002	[−0.014, −0.006]	−0.016	0.009	[−0.033, 0.001]
Model Fit						
*R^2^*	0.600			0.594		
Δ*R^2^*	0.008			0.002		
*F* change	22.189			3.566		
Conditional effects of coping at values of GSE						
Low GSE (16^th^ percentile)	0.296	0.031	[0.235, 0.357]	—	—	—
Mean GSE (50th percentile)	0.225	0.026	[0.173, 0.276]	—	—	—
High GSE (84th percentile)	0.133	0.031	[0.072, 0.195]	—	—	—

*N* = 1,937. B, unstandardized coefficient; SE, standard error (HC3); CI, confidence interval; GSE, general self-efficacy. Coping and GSE were mean-centered prior to analysis. Conditional effects are not reported for avoidant coping because the interaction was not statistically significant (*p* = 0.059).

## 4 Discussion

This study examined the associations between coping strategies, GSE, and mental wellbeing among TVET trainees and investigated the moderating role of GSE in the coping–wellbeing relation. It tested four hypotheses, three of which received full empirical support while one was partially supported, yielding a theoretically coherent and practically meaningful pattern of findings.

Consistent with H1, approach coping demonstrated a strong positive association with mental wellbeing, whereas avoidant coping showed no meaningful bivariate association. This is theoretically coherent with Lazarus and Folkman's (1984) framework, in which approach-oriented strategies are most adaptive when a stressor is appraised as controllable, a condition plausibly met within the TVET environment, where trainees' effort and strategy substantially shape performance outcomes. It also aligns with findings suggesting that active, goal-directed coping, characterized by effortful engagement with the stressor through planning, information-seeking, and instrumental action, is most strongly associated with positive psychological outcomes (Kwon, 2025; Waterhouse and Samra, 2025). The breadth of the approach dimension, which encompasses both instrumental strategies and emotion-regulatory ones such as seeking social support, acceptance, and meaning-making, indicates that both forms of engagement contribute to wellbeing. This is consistent with Theodoratou et al. (2023) and Perry and Cuellar (2021), who emphasized that positive emotion generation allows individuals to mobilize social support and engage in activities that provide relief from stressors and further recommended that institutions embed such mechanisms within everyday practices to support learners during periods of vulnerability.

Avoidant coping showed a significant negative association once approach coping was controlled (*B* = −0.183, *p* < 0.001), but had a negligible zero order association with mental wellbeing (*r* = 0.073). This pattern was a consequence of statistical suppression, since the two coping dimensions were moderately correlated (*r* = 0.397). Trainees who coped more actively also reported slightly higher on avoidance and the mutual variance between the two dimensions overshadowed the negative influence of avoidance on the coping scale. After this overlap is accounted for, the association is consistent with the predominant characterization of avoidant coping as maladaptive, with behavioral disengagement and denial, substance abuse and psychological maladjustment linked to poor mental wellbeing (Chaaya et al., 2025; Rodríguez et al., 2026). The effect of this unique response was, however, small, suggesting that responses toward disengagement had a small negative relationship with wellbeing in this sample.

Notably, confirmatory factor analysis revealed that the items of self-distraction did not load into the avoidance dimension in this sample but rather functioned as approach coping strategies; focusing on work or other activities, reading, or watching television, grouped with active coping, planning, and seeking support, instead of denial or disengagement. This might reflect the structure of competency-based education and training in TVET, where redirecting effort into the practical workshop activity is both available and productive, and distraction is therefore not a withdrawal from stressor but an engagement with another task. This finding is consistent with evidence that disengagement is not uniformly maladaptive and that some forms of distraction may be constructive (Waugh et al., 2020), and it contributes to a limited evidence base on the structure of the Brief-COPE in African TVET settings.

Findings partially supported H2, with approach coping demonstrating a strong positive association with GSE, whereas avoidant coping showed no significant association. Consistent with Bandura's (1977) self-efficacy theory, these results position self-efficacy beliefs as both determinants and outcomes of adaptive behavior. As observed in prior findings, TVET trainees with higher self-efficacy are more likely to approach stressful situations with greater confidence and perceive their capacity to manage demands and are therefore more inclined to implement active, approach-oriented coping strategies (Freire et al., 2020; Morales and Pérez-Mármol, 2019) whereas those with lower self-efficacy may not be able to adapt to increasing academic demands and thus tend to rely on maladaptive strategies such as procrastination, denial, and disengagement (Choi et al., 2026). The absence of any association between avoidant coping and self-efficacy is itself informative: efficacy beliefs related strongly to engagement-oriented coping and not at all to disengagement, supporting the treatment of these dimensions as distinct constructs rather than opposite poles of a single construct.

GSE demonstrated a large positive association with mental wellbeing, strongly supporting H3. This aligns with findings on the positive association between self-efficacy and psychological wellbeing across diverse populations (Alotaibi and Awed, 2025; Bandura, 1977; Grotan et al., 2019; Sun et al., 2025); the present study observed this relation within an under-researched cohort. TVET trainees with high efficacy beliefs tend to embrace academic challenges with confidence, persevere amid difficulties, and recover from failure, all of which help maintain positive emotions and minimize distress. This phenomenon is relevant because these trainees face the cognitive and practical challenges of vocational training; thus, belief in one's capability is a strategy that highlights self-efficacy as an important psychological resource for supporting wellbeing.

The moderation analyses provided this study's most theoretically distinctive findings. GSE significantly moderated the relation between approach coping and mental wellbeing, with a negative interaction indicating an attenuating pattern. The approach-coping wellbeing benefit was strongest among trainees with low self-efficacy (*B* = 0.296) and gradually declined as self-efficacy increased (*B* = 0.133 at the 84th percentile), though it remained significant across the entire observed range. The corresponding interaction for avoidant coping was not significant (*p* = 0.059), indicating that the attenuating role of self-efficacy was specific to engagement-oriented coping. It's worth acknowledging the magnitude of moderation; the interaction accounted for less than 1% of additional variance in mental wellbeing (Δ*R*^2^ = 0.008). In raw terms, a 10-point difference in approach coping corresponded to approximately 3.0 additional WEMWBS points among trainees at the 16th percentile of self-efficacy, compared with 1.3 points at the 84th percentile. The moderation was statistically robust and detectable in a large sample, but modest in absolute magnitude, and therefore it was characterize as attenuating the contribution of coping to wellbeing rather than substituting for it.

Approach coping consistently significantly associated with wellbeing across all self-efficacy levels, with Johnson-Neyman analysis identifying no transition point within the observed range. This underscores the role of engagement-oriented coping as a basic adaptive resource benefiting trainees irrespective of efficacy beliefs, consistent with Lazarus and Folkman's (1984) assertion that problem-focused coping is the most fundamentally adaptive response to controllable stressors. Together, these findings extend the understanding of the coping–wellbeing relation by demonstrating that self-efficacy is not simply a correlate of coping and wellbeing but also a moderator of the relation between the two, highlighting the importance of studying the interactive effects of psychological resources (Guillot-Valdés et al., 2026; Woreta et al., 2025).

The current findings have several practical implications regarding the provision of wellbeing support in TVET institutions. The positive association between approach coping and mental wellbeing highlights the importance of integrating structured coping-skills development as part of the induction and training support given to trainees, with emphasis on engagement-oriented strategies given their consistent links with both wellbeing and self-efficacy. Most distinctively, the moderation findings inform support that targets low-self-efficacy trainees as coping-based interventions yielded the greatest wellbeing benefits in this group. Therefore, by screening for low self-efficacy at entry, those most at risk and most likely to benefit from coping-skills training would be identified. For trainees with already high self-efficacy, support may be more productively oriented toward maintaining existing efficacy resources than building coping repertoires.

Several limitations should be considered when interpreting these findings. First, and most fundamentally, the cross-sectional design does not allow for causal inference. Although self-efficacy was modeled as a moderator and coping as a predictor, the temporal ordering of these relations cannot be established, and the associations are equally consistent with reciprocal influence, in which trainees who cope effectively develop stronger efficacy beliefs over time. Therefore, longitudinal designs must be adopted to clarify the direction of these relations and directly test the resource-attenuations interpretation. Second, all constructs were assessed using self-report instruments, which introduce the potential for common method variance and social desirability bias. Although Harman's single-factor test suggested that common method variance was not a significant source of the observed relations, this study's sole reliance on self-report remains a constraint; hence, future research should incorporate multi-source data collection measures of coping and wellbeing to strengthen the findings.

Third, the hypothesized three-dimensional structure of the Brief-COPE was not supported in this sample. Problem-focused and emotion-focused coping were empirically indistinguishable (latent *r* = 0.982). The two-factor approach–avoidance solution was therefore adopted, which provided an acceptable fit and clear factor separation. This structure was specific to this context; the approach dimension was broad combining strategies of differing adaptiveness, with self-blame and venting grouped alongside active coping and planning. A single association with wellbeing may therefore mask variation among these components. Fourth, trainees were sampled within institutions, but institutional identifiers were not retained in the dataset, recording only institution type. It was therefore not possible to estimate the intraclass correlation or apply cluster-robust standard errors. Unmodelled clustering may have resulted in underestimated standard errors, and given the modest magnitude of the interaction effects, multilevel replication with retained institutional identifier is a priority for future work. Fifth, although sociodemographic characteristics were entered as covariates and the moderation effect proved robust to their inclusion. However, institutional type was modeled as a single ordinal indicator rather than through coding, and unmeasured institutional characteristics such as staffing ratios or counseling provision were also not available. Therefore, further research that explicitly examines contextual and demographic factors would facilitate the generalization of findings to other TVET systems and trainee populations.

## 5 Conclusion

This study conceptually and empirically contributes to the understanding of TVET trainees' mental wellbeing by showing that coping strategies and GSE function as psychological resources that interact to collectively influence mental wellbeing in TVET settings. The moderating role of self-efficacy in the relation between approach-oriented coping and mental wellbeing highlights self-efficacy as not only a contributor to wellbeing outcomes but also a moderator that shapes how strongly coping relates to wellbeing, such that those with the lower self-efficacy beliefs gain the most. This moderating role was specific to engagement-oriented coping and was modest in magnitude, and the benefits of approach coping remained significant at all levels of self-efficacy. Overall, self-efficacy is a key psychological resource that facilitates trainee wellbeing in TVET institutions, with implications for targeted, evidence-based interventions in the design of such institutions. Future longitudinal and cross-demographic research should be conducted to establish causal directionality and extend the generalizability of these findings across diverse TVET learning environments and trainee populations.

## Data Availability

The raw data supporting the conclusions of this article will be made available by the authors, without undue reservation.
