# Supplementary material for: Coping strategies and mental wellbeing among TVET students: the moderating role of self-efficacy
Source: Front Psychol. 2026 Oct 20;17:1951147. doi: 10.3389/fpsyg.2026.1951147 (PMC13727609; doi:10.3389/fpsyg.2026.1951147)
Supplement: Supplementary file 1 [file Table_1.docx]

**Table S1***.*

Brief-COPE item mapping

| **Variable** | **Item wording** | **Brief-COPE facet** | **Dimension** | **λ** |
| --- | --- | --- | --- | --- |
| COPE1 | I turn to work or other activities to take my mind off things. | Self-distraction | Approach | .679 |
| COPE2 | I concentrate my efforts on doing something about the situation. | Active coping | Approach | .691 |
| COPE3 | I say to myself, "this isn’t real." | Denial | Avoidant | .554 |
| COPE4 | I use alcohol or other substances to help me get through it. | Substance use | Avoidant | .584 |
| COPE5 | I get emotional support from others. | Emotional support | Approach | .570 |
| COPE6 | I give up trying to deal with it. | Behavioral disengagement | Avoidant | .667 |
| COPE7 | I take action to try to make the situation better. | Active coping | Approach | .696 |
| COPE8 | I refuse to believe that it has happened. | Denial | Avoidant | .589 |
| COPE9 | I say things to let my unpleasant feelings escape. | Venting | Approach | .542 |
| COPE10 | I use alcohol or other substances to make myself feel better. | Substance use | Avoidant | .602 |
| COPE11 | I try to get comfort and understanding from someone. | Emotional support | Approach | .633 |
| COPE12 | I give up the attempt to cope. | Behavioral disengagement | Avoidant | .674 |
| COPE13 | I look for something good in what is happening. | Positive reframing | Approach | .724 |
| COPE14 | I make jokes about it. | Humor | Approach | .496 |
| COPE15 | I do something to think about it less, such as watch TV, read, or sleep. | Self-distraction | Approach | .686 |
| COPE16 | I accept the reality of the fact that it has happened. | Acceptance | Approach | .717 |
| COPE17 | I express my negative feelings. | Venting | Approach | .446 |
| **COPE18** | I turn to work or other activities to take my mind off things. [2] | Duplicate of COPE1 | ***EXCLUDED*** | *—* |
| COPE19 | I try to find comfort in my religion or spiritual beliefs. | Religion | Approach | .710 |
| **COPE20** | I try to learn something from the experience. | Not a Brief-COPE item | ***EXCLUDED*** | *—* |
| COPE21 | I focus on getting advice or help from others. | Instrumental support | Approach | .736 |
| COPE22 | I use humor to cope with the situation. | Humor | Approach | .634 |
| COPE23 | I learn to live with it. | Acceptance | Approach | .674 |
| COPE24 | I think hard about what steps to take. | Planning | Approach | .671 |
| COPE25 | I blame myself for things that happened. | Self-blame | Approach | .350 |
| COPE26 | I pray or meditate. | Religion | Approach | .730 |
| COPE27 | I seek help and advice from others. | Instrumental support | Approach | .713 |
| COPE28 | I try to see it in a different light, to make it seem more positive. | Positive reframing | Approach | .748 |

*Note:* Items listed in the order they appear in the dataset. Standardized loadings are from the retained two-factor confirmatory factor model.

**Summary: Approach coping** = 20 items (α = .934): active coping, planning, instrumental support, emotional support, positive reframing, acceptance, religion, humor, venting, self-blame, self-distraction. **Avoidant coping** = 6 items (α = .793): denial, substance use, behavioral disengagement. Factor correlation = .410. Two items were excluded: COPE18 duplicates COPE1 verbatim, and COPE20 is not a Brief-COPE item. Their exclusion means one planning item and one self-blame item from the published instrument are absent from this administration. Following the exclusion of COPE18 and COPE20, planning and self-blame facets are each represented by a single item - COPE24 and COPE25 respectively, while all other facets retain both of their items.

**Note on the correction.** Subscale scores were originally computed using published item numbering (Carver,1997), which did not correspond to the item order of the administered instrument. All scales were recomputed from the facet assignments shown above. Under the published numbering, problem-focused scale contained substance-use and behavioral-disengagement items and avoidant scale contained emotional-support, acceptance and religion items.
